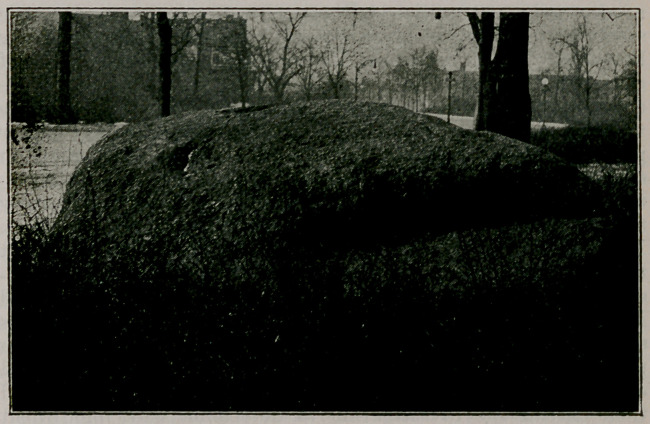# Topics of Public Interest

**Published:** 1917-08

**Authors:** 


### TOPICS OF PUBLIC INTEREST

**Centenarians.** Il Policlinico, Rome, Feb. 18, notes the death of Mmme. Rosa Posseti at Tarente, aged 107. Until a few years ago, she conducted a shop for the sale of wood. La Revista Medico Ocirurgica of Brazil, Nov. 1916, reports the death of Jose Fernando Guimaraes, born in Portugal in 1775, who died Oct. 13, 1916. Up to the age of 85, he was a shoemaker; he then took employment as helper in a store. Memory and sight were perfect till recently. The Vicount of Barbacena, robust and active at the age of 104 is also cited. Mrs. Louisa K. Thiers of Milwaukee, a literal daughter of the Revolution, aged 101 is among the purchasers of Liberty Bonds.

---

**Increase in High Rank Positions in the Army Medical Corps.** Senator Owen has introduced an amendment providing that the same proportionate distribution shall be made as for the Navy. This means ½% generals, 4% colonels, 8% lieutenant colonels. With the regular army increased to 293,000 (act of June 3, 1916), the medical department will have about 2000 officers. Last year, it numbered 604: 1 general (regularly a brigadier, Dr. Gorgas having been made a major general for his distinguished services in the Panama Canal sanitation), 14 colonels, 24 lieutenant colonels, 105 majors, 239 captains and 221 first lieutenants. The new allotment will provide for 9 brigadier generals, 80 colonels and 160 lieutenant colonels, will allow promotion of one or two grades for most of the officers holding commissions in 1916 so that nearly all members of the department at that time will be at least majors.

---

**New Poison Gas.** La Suisse reports that the Germans have devised a new gas, smokeless and odorless and hence difficult to detect. Those inhaling it notice at first only a sweetish taste but it is very fatal and troops employing it have to wear masks continuously.

---

**Transfer From Public Health Service to Navy.** 17 surgeons of the former service have been transferred to duty with the Navy.

---

**Dr. Samuel Guthrie.** In the May and June issues, were published a biography prepared by Dr. W. V. Ewers of Rochester. Portraits here presented are of Mr. T. S. Chamberlin and Mr. F. C. Chamberlin of Chicago, respectively grandson and great grandson of the late Dr. Guthrie. These are said by a lady now over 90 years old, to give a very good idea of the personal appearance of Dr. Guthrie at the corresponding ages. The former, she declares to be an exact likeness of Dr. Guthrie as she knew him. The Guthrie bowlder stands at the entrance of Washington Park, Chicago, from Garfield Bvd., originally 55th St.

---

**Million Acre Wheat Campaign for N. Y.** A committee under the auspices of the N. Y. State Agricultural Society has begun this campaign and will supply seed and fertilizer on credit. In 1879, 736,000 acres were devoted to wheat in N. Y. 30 years later, the average was 289,000. The total area of the state is about 32 million acres. The average yield of winter wheat in N. Y. is a trifle over 20 bushels per acre, as compared with a trifle less than 12 bushels of spring wheat in N. Dakota, a typic spring wheat state. This year, it is estimated that N. Y. will produce 9 million bushels of wheat on 430,000 acres. The home consumption of wheat for N. Y. State, including N. Y. City, is about 60 million bushels, requiring about 3 million acres, or about 1/10 of the total area of the state and considerably more than 1/10 of the arable land. From one point of view, it would be ideal to have N. Y. self-supporting as to bread stuffs but there are numerous practical points to consider. For example, milk as such, is equally needed and there are obvious reasons why it should be produced as near as possible to centers of consumption. Dairy products are also highly desirable and they have increased in price even more markedly than wheat and flour. Grains in general are practically free from serious problems as to preservation and transportation and the whole matter of food supply, must be worked out with reference to availability of fertilizer, avoidance of exhaustion of soil, labor supply, economy of transportation, etc., for the whole country. According to the estimate of June 6, the total wheat crop of the U. S. this year will be 656 million bushels, leaving about 36 million for export, whereas the Allies will need nearly 300 million bushels. From a narrow, domestic standpoint, the problem of wheat necessity could very easily be solved by using oatmeal for breakfast and some form of corn bread for one other meal. But, while oats and corn are commonly regarded as cheap cereals, the practical obstacle remains that they ordinarily retail at considerably higher prices than wheat flour and are still somewhat higher, in spite of the extraordinary demand for wheat and the agitation regarding economy of food stuffs. The same is generally true of other cereals that might be used as wheat substitutes. If we extend the investigation to vegetables generally, that may be considered as more or less available as wheat substitutes, the same practical economic problem is encountered in even greater degree. It occurs to us that food commissions should give their immediate attention to the oats and corn problem and determine whether the obstacle of price is based on essential economic factors or on other factors and should then extend the investigation to determine why fine wheat flour, refined sugar and even some tropical and semi-tropical fruits are relatively economic as compared with what are ordinarily considered cheaper grains, leguminous seeds, fresh vegetables and domestic fruits.

---

**Notes on Early History of Buffalo Medical Journal,** contributed by Librarian of The New York Academy of Medicine. Buffalo Medical Journal, Edited by Austin Flint, (monthly), vols. 1-15, June, 1845 to June, 1860. Buffalo. Vol. 2-15, 1846-60, Title Buffalo Medical Journal and monthly review of Medicine and Surgical Science; vol. 15, 1859-60, running title in New York Medical Review of Medical and Surgical Science and Buffalo Medical Journal; it contains 13 Nos., June, 1859 to June, 1860; vol. 9, 1853-4, Sanford B. Hunt added as editor; vol. 11-12, 1855-1857, Dr. Hunt sole editor; vol. 13, 1857-58, Austin Flint, Jr., added; vol. 14, 1858-59, Dr. Hunt dropped; No. 10, vol 15, March, 1860, Dr. Douglas added; in July, 1860, merged in American Medical Monthly; vols 1-18, 1854-62, Buffalo Medical Journal and Monthly Review of Medical and Surgical Science; title of vol. 2-15, 1846-60 of Buffalo Medical Journal; Buffalo Medical and Surgical Journal, Edited by Julius F. Miner; (monthly), vol. 1-28, August, 1861-89; title of vol. 1, 1861-2, Buffalo Medical and Surgical Journal and Reporter; vol 13, 1873-74, Edward N. Busch added as editor; vol. 17, 1878-9, W. W. Miner added; in vol. 19, 1879-80, Thos. Lothrop, Herman Mynter, A. R. Davidson, P. W. Van Peyma and Lucien Howe became editors; Dr. Miner died Nov. 5, 1886.

---

**Military Rank for Red Cross Workers.** Assimilative military rank, with authority to use the proper titles, insignia and uniforms, is authorized by the War Dept. for all members of the Red Cross. This authorization is extended both as a courtesy and appreciation of services and to insure proper respect for the individuals by belligerent troops of all nationalities involved. It does not imply payment by the government nor responsibility for employment. The ranks range from that of major-general for the chairman of the war council, through all grades of commissioned and non-commissioned officers to private. Directors of camps, base hospitals, supply depots, etc., will be graded as majors, the assistant surgeons in these units being captains and first lieutenants. Officers of the Red Cross will also wear the Greek cross in red enamel, as the predominating mark of the service.

---

**Military Vaccination.** American soldiers, going to Europe, will be vaccinated against small pox, typhoid and paratyphoid A and B.

**Poison Gases.** The asphyxiating and lachrymating gases depend to a considerable degree upon sabadilla seeds. These contain cavadine and sabadilline, alkaloids and veratric acid. The Venezuelan supply was largely bought up by Germany before the war. A wild plant of the same genus is found in Texas and it is believed that this could be used by the allies. (Note: Without expressing a personal preference, we hope that the U. S. and its allies will secure a successful termination of the war by the means ordinarily employed in war for the last few centuries, with due allowance for improvements along established lines).

---

**Filtration Plants.** In 1900, less than 2 million inhabitants of the U. S. were provided by municipalities with filtered water. At present, 20 million are thus protected, in 781 cities and town.

---

**Wheat, Corn and Oats.** Here is a practical dietetic problem for the Food Director. The official Bulletin of the U. S. publishes some very interesting statistics of export for the 11 months ending with May 1917. For our present purpose, it is significant that wheat was exported at an average price of a little less than $2 a bushel of 60 pounds, or at about 3¼ cents a pound; flour at almost exactly $8 a barrel or 4 cents a pound. Corn was exported at about $1.08 a bushel of 56 pounds, a trifle less than 2 cents a pound. Oats was exported at a trifle over 60 cents a bushel of 32 pounds, or a trifle less than 2 cents a pound. Export values are about as good an index of true values as can be obtained. The people of the U. S. are urged to use other cereals to replace wheat so far as practical and it would be a perfectly simple matter, involving no sacrifice either of nutritive value—except for a rather recondite allowance for variations in aminoacids in favor of wheat—or of palatability for practically every one to use some form of oatmeal for breakfast and some form of corn bread for one other meal, thus using wheat for only a third of the cereal nutrition and actually reducing the consumption of wheat for domestic purposes to about half the customary amount and liberating this amount for the Allies. But, here is a practical economic obstacle: Approximately and with due allowance for fluctuations in price of the raw and finished domestic product which have been marked in the last few months, the ultimate consumer pays about 1/3 more for flour than the unground wheat is worth wholesale. On the other hand, he pays 2/3 times as much for cornmeal as the unground corn is worth and 4/5 times as much for "oatmeal" which is usually not a meal at all, as oats are worth wholesale in the crude state. At present, with a promise of a reduction of wheat flour to about 6 cents a pound in the near future, we are paying not quite 8 cents; while cornmeal costs 6 cents and ''oatmeal'' 8 or 9 cents, according to the special preparation employed. We appreciate the fact that special, fancy preparations of cereal of any kind are expensive but we do not see why the elaboration of one grain into a flour or meal or other domestic product, should be materially different from that of any other, especially at a time when there are adequate economic reasons for the high price of wheat and wheat flour and patriotic reasons for replacing them with other cereals, usually considered cheaper and still actually and relatively so in the wholesale market.

---

**Inaccuracy of Auditory Sensations.** While human beings have communicated thought for many milleniums by speech, implying the discrimination of elementary vowel and consonant sounds, any one who has given attention to the matter realizes that phonetic speech changes considerably in the course of a few generations, that the actual pronunciation of the same word differs considerably for different individuals and, often without realization of the fact, from time to time in the same individual. Every little while a story is repeated in the press, with differences in local color, to the effect that a stenographer takes down in phonetic short hand, foreign speech which he does not understand. Most stenographers brand this story as a lie but it is worth while to inquire whether or not there are exceptionally acute ears which really do appreciate elementary speech sounds accurately. Recently, we have had our attention called to this matter by the repeated mistake of ''Central'' in understanding three as six. The two words do not have any sound in common and they differ in regard to number and arrangement of elementary sounds about as much as any two short words can. It is difficult to explain how even the most slovenly pronunciation of either word could approximate that of the other. Can any student of otology throw light on the confusion?

---

**Exaggeration of War Losses of Physicians.** Sensational statement have been made, as that 60,000 British physicians had been lost in the present war. As the entire population of the British Islands is only about 46 millions and the entire British population of the various other components of the Empire, scarcely as much more, it is probable that, aside from native physicians more or less qualified, who have not participated in the War to any great degree, there were not much more than 60,000 British physicians altogether, at the outbreak of the War. At any rate, only about 12,000 have been employed in military duty and, up to June 25, the total losses for the duration of the War, were: 195 killed, 62 died of sickness, wounded 964. The deaths, with ample allowance for the remainder of the third year of the war, amount to 7 or 8 per thousand, per year. The expected deaths, in peace, for males of the ages covering active military service by physicians, amount to about 5 per thousand per year. In other words, not counting non-fatal wounds, the military risk amounts to about half of the inevitable risk of death under peace conditions. Yet it is said that the risk has been greatest for medical officers and greater for officers in general than for privates.

---

**Warning Against Burma Bean..** The U. S. Dept. of Agriculture calls attention to the fact that this bean has been shipped into the country via Canada, from the east. It resembles the ordinary navy bean (so-called because really used largely in the navy) but can be distinguished by the light yellow color and the presence of fine lines radiating from the hilum. They will not grow well except in the tropics and this is fortunate for they contain too much hydrocyanic acid to render them safe for food.

---

**High School Graduates, Buffalo, 1917:**

| | Boys. | Girls. |
|---|---|---|
| Hutchinson-Central | 79 | 103 |
| Masten Park | 63 | 87 |
| Lafayette | 110 | 130 |
| Technical | 82 | 9 |
| South Park | 14 | 14 |
| Total | 248 | 343 |

For any single age-year, at the average of graduation, about 2% fairly equally divided as to sex, would be eligible. Thus about 7% or 1 person in 14 of the rising generation in Buffalo, has a full high school training. In some states, the ratio reaches 10%.

---

**The N. Y. State Narcotic Law** has been amended and nominally takes effect July 1 but, owing to pressure of clerical work, has been postponed till Aug 1. All physicians should secure a copy of the law and read it and save it for reference. It is published in the Official Bulletin of the State Dept. of Health and may be obtained on application to Dr. Hermann M. Biggs, Health Commissioner, Albany.

**N. Y. Medical Journal Ambulance.** The A. R. Elliott Publishing Co. has presented a fully equipped Ford ambulance, through the Montclair N. J. Chapter of the Red Cross. We congratulate the company on its patriotism and wish the finances of this journal warrant a like contribution.

---

**Gift of Ambulances.** The Ford Motor Co. has voted to give $500,000 worth of ambulances—about 1,000 to the Red Cross.

---

**Centenarian.** Mrs. Abbie Maria Harris died near Rome, July 7. She was born in the vicinity, Dec. 13, 1814.

---

The most modern hospital train to be found in this country or abroad has been presented to the state of Maryland by three railroads, the Baltimore and Ohio, the Pennsylvania and the Western Maryland. There are six cars which make up the train. Three of these are hospital cars, with forty-two hospital beds; an operating car, which it is claimed is not surpassed in the completeness of its equipment by any operating room in the city; a Pullman and dining car for the personnel of the hospital, and an express car which carries two motor ambulances. An overhead trolley by means of which a stretcher may be carried from one car to another is a special feature.

---

**Automobiles for Military Purposes.** We have good reason for stating, though without implying that the statement is authorized or that future emergencies may not change the conditions, that there will be no commandeering of automobiles and, as a corollary, that no patriots will have the opportunity to sell second-hand automobiles to the government. The factories can supply all that will be needed and the needs for the present war, under existing and probable future conditions, will not be excessive. There will be no spectacular charge of a light brigade in Fords.

---

**Military Service for Lunatics.** According to the Associated Press, the annual report of a German Lunatic Asylum at Settin, states that lunatics have proved unsatisfactory, on account of desertions, refusal to obey orders, and general lack of discipline. (Note: There can be no issue of opinion on either the question of efficiency or ethics. The real point at issue, as in regard to rendering corpses, is whether the report is true or garbled.)

---

**Statistics of Women Physicians.** Dr. Mary Sutton Macy of N. Y., Med. Rec., Apr. 21, has compiled statistics from the

Am. Med. Directory of 1916, judging sex by Christian names, not absolutely an accurate method on account of the frequent use of names that might be applied to either sex. Christian names, usually considered male, have been found among the alumnae of women's colleges and vice versa. The proportionate number of women varied from 0.47% for Alabama to 8.09% for Massachusetts. The percentages of women physicians connected with state societies varied from 0 for Delaware and Nevada (totals 6 and 5, respectively) to 69 for S. C. (total 13), and averaged 38.08 against 53.72 for men. The total number of physicians in the Continental U. S. is 145,-240, women 5,518; including dependencies, 146,612 and 5,551 respectively. The distribution of the profession by states and of women by specialties is shown in the following tables.

| Specialty | Practised Exclusively by | Special Interest Expressed by | Total of Medical Women Specializing by |
|---|---|---|---|
| Gynecology | 53 | 466 | 519 |
| Obstetrics | 9 | 134 | 143 |
| Pediatrics | 10 | 119 | 129 |
| Surgery | 11 | 63 | 74 |
| Ophthalmology, otology, laryngology and rhinology | 42 | 29 | 74 |
| Neurology | 15 | 51 | 66 |
| Psychiatry | 21 | 36 | 57 |
| Ophthalmology | 24 | 25 | 49 |
| Clinical pathology | 14 | 20 | 34 |
| Anesthesia | 6 | 22 | 28 |
| Otology, laryngology and rhinology | 8 | 17 | 25 |
| Pathology | 9 | 10 | 19 |
| Dermatology | 6 | 10 | 16 |
| Roentgenology | 5 | 11 | 16 |
| Tuberculosis | 3 | 13 | 16 |
| Orthopedic surgery | 1 | 9 | 10 |
| Internal Medicine | 8 | 0 | 8 |
| Laryngology and rhinology | 1 | 7 | 8 |
| Public health | 7 | 1 | 8 |
| Bacteriology | 4 | 3 | 7 |
| Ophthalmology and otology | 1 | 4 | 5 |
| Proctology | 0 | 3 | 3 |
| Urology | 0 | 2 | 2 |
| Totals | 258 | 1,055 | 1,313 |

| State | Total Physicians (A. M. A. Figures) | Women Physicians (Approximate) |
|---|---|---|
| Alabama | 2,569 | 12 |
| Arizona | 307 | 9 |
| Arkansas | 2,637 | 25 |
| California | 5,687 | 520 |
| Colorado | 1,733 | 111 |
| Connecticut | 1,678 | 63 |
| Delaware | 261 | 6 |
| District of Columbia | 1,482 | 59 |
| Florida | 1,321 | 33 |
| Georgia | 3,421 | 28 |
| Idaho | 438 | 11 |
| Illinois | 10,648 | 657 |
| Indiana | 4,872 | 169 |
| Iowa | 3,751 | 170 |
| Kansas | 2,683 | 114 |
| Kentucky | 3,584 | 70 |
| Louisiana | 2,060 | 23 |
| Maine | 1,205 | 36 |
| Maryland | 2,292 | 74 |
| Massachusetts | 5,869 | 475 |
| Michigan | 4,360 | 176 |
| Minnesota | 2,447 | 96 |
| Mississipi | 2,048 | 18 |
| Missouri | 6,399 | 158 |
| Montana | 636 | 22 |
| Nebraska | 1,911 | 98 |
| Nevada | 154 | 5 |
| New Hampshire | 690 | 35 |
| New Jersey | 3,239 | 152 |
| New Mexico | 430 | 9 |
| New York | 15,670 | 710 |
| North Carolina | 2,102 | 22 |
| North Dakota | 586 | 13 |
| Ohio | 8,045 | 301 |
| Oklahoma | 2,634 | 54 |
| Oregon | 1,187 | 86 |
| Pennsylvania | 11,502 | 482 |
| Rhode Island | 772 | 26 |
| South Carolina | 1,399 | 13 |
| South Dakota | 676 | 19 |
| Tennessee | 3,457 | 30 |
| Texas | 6,240 | 81 |
| Utah | 465 | 16 |
| Vermont | 668 | 12 |

| | | |
|---|---|---|
| Virginia | 2,547 | 20 |
| Washington | 1,695 | 77 |
| West Virginia | 1,729 | 30 |
| Wisconsin | 2,803 | 79 |
| Wyoming | 251 | 14 |
| Alaska | 86 | 5 |
| Canal Zone | 97 | 3 |
| Hawaii | 162 | 2 |
| Philippine Islands | 735 | 17 |
| Porto Rico | 292 | 6 |

**Deaths from Automobiles** for the first half of 1917, numbered 302 for N. Y. State, 160 for the city, 142 for the rest of the state.

**German Birth Rate.** 26 cities of 200,000 and over report an actual decrease of about 98,000 births, or 38.3% as compared with 1914 and the decrease for all cities of over 15,000 is 39.5%. (Such a fall is to be expected under present circumstances, for all belligerents, and is probably to be regarded as conservative. The destruction of property will be less severely felt if there are fewer persons among whom it is to be distributed and while the actual use of property of any kind is small by very young children, their presence is a considerable drawback in times of need, such as accompanies and immediately follows war.)

**Typhoid Epidemic.** The town of Orillia, Ont., in 3 weeks up to May 17, had 140 cases in a population of 8,000. In the same period, Toronto had 1 case in a population of 500,000. The water supply was not at fault and the epidemic was traced to milk.

**Nutritional Value of Exports.** A government statistician has estimated that the exports from the U. S. for a year, at the rate of the first nine months of the fiscal year from July 1 to June 30, 1917, would feed 17,686,000 persons for a year, on the Voit ration of 118 grams of protein, 500 of carbohydrate and 56 of fat, with a surplus of over 2¼ billion pounds of carbohydrate and 429 million pounds of fat. On the Chittenden ration of 60 grams of protein, 500 of carbohydrate and 56 of fat, 54,783,000 persons could be fed but there would be a deficiency of more than 4½ billion pounds of carbohydrate and 314 million pounds of fat. On the basis of an allowance of 3,055 calories, without specification, 20,388,000 persons could be fed. This allowance is high and as the proteid covers a considerably larger number on the lowproteid standard and as the Voit protein standard is higher than is necessary, even if we do not concede Chittenden's claims to their full extent, it is fair to estimate that our exports provide for 25,000,000 persons a year.

## Figures and Tables

**Figure f1:**
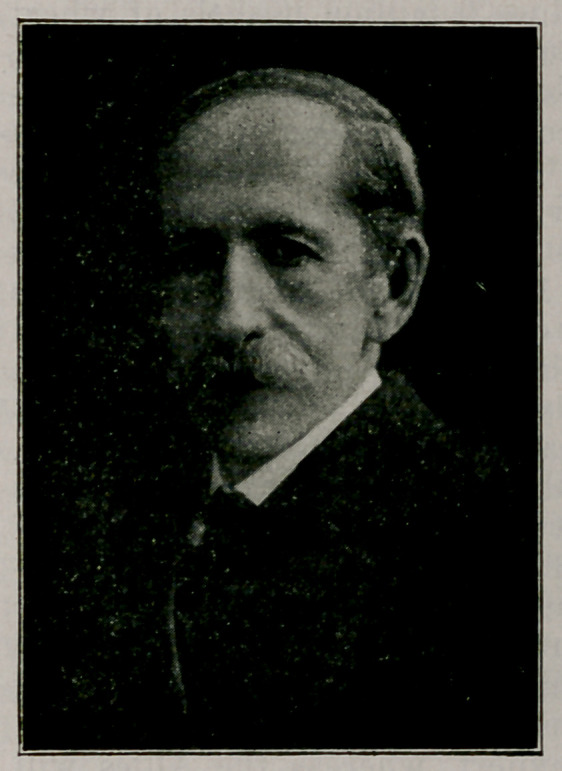


**Figure f2:**
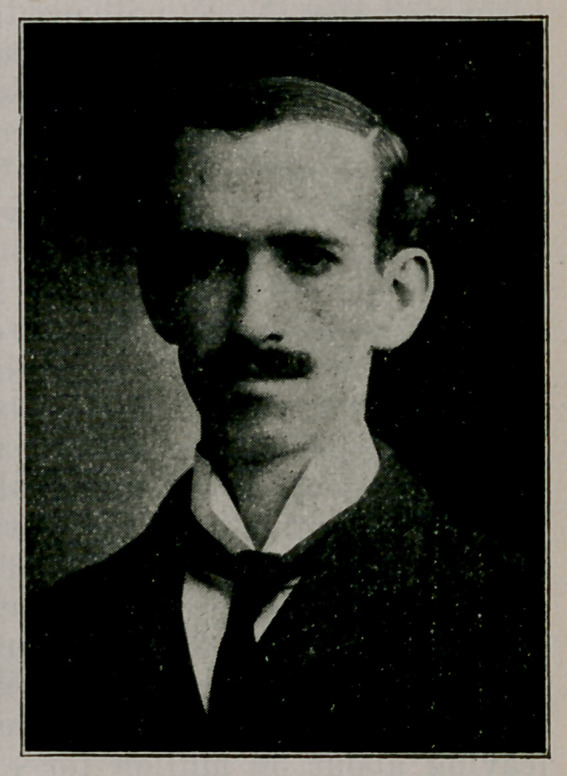


**Figure f3:**